# Vitamin D stimulates Il-15 synthesis in rodent muscle

**DOI:** 10.1016/j.bbrep.2025.101925

**Published:** 2025-01-25

**Authors:** Franz Ewendt, Fabienne Drewitz, Michael Althammer, Cosima Eichler, Corinna Brandsch, Stefanie Brey, Thomas H. Winkler, Mirja R. Wilkens, René St-Arnaud, Marina Kreutz, Gabriele I. Stangl

**Affiliations:** a Martin Luther University Halle-Wittenberg, Institute of Agricultural and Nutritional Sciences, 06120, Halle (Saale), Germany; b Institute of Functional Genomics, University of Regensburg, Regensburg, Germany; c Division of Genetics, Department Biology, Friedrich-Alexander University Erlangen-Nürnberg, 91058, Erlangen, Germany; d Institute of Animal Nutrition, Nutrition Diseases and Dietetics, Faculty of Veterinary Medicine, University of Leipzig, Leipzig, Germany; e Shriners Hospitals for Children - Canada and McGill University, Montréal, Quebec, Canada

**Keywords:** Calcitriol, Immune system, Cytokine, Vitamin D receptor, Muscle-immune-crosstalk, Immunosenescence

## Abstract

Besides its classical skeletal function, vitamin D plays a critical role in both skeletal muscle and the immune system. Interleukin-15 (IL-15), which is highly expressed, and secreted complexed with its receptor, IL-15Rα, by skeletal muscle, stimulates the development of immune cells and affects myogenesis and muscle mass. However, little is known about possible regulators of this myokine. To test whether vitamin D could be a regulator of muscle IL-15 and IL-15Rα expression, C2C12 myotubes were treated with vitamin D_3_ metabolites and analysis were performed in gastrocnemius muscles of rats treated with a single intraperitoneal dose of 1,25(OH)_2_D_3_. The role of VDR was investigated by siRNA technique in C2C12 myotubes and in gastrocnemius muscles of vitamin D receptor knockout (Vdr-KO) mice. Treatment of C2C12 myotubes with 1,25(OH)_2_D_3_ or 25(OH)D_3_ increased *Il-15* gene expression in a dose-dependent manner and 1,25(OH)_2_D_3_ also moderately increased the relative Il-15 protein amount. Rats treated with a single dose of 1,25(OH)_2_D_3_ demonstrated a higher mRNA abundance of muscle *Il-15* than controls. The 1,25(OH)_2_D_3_ effect on *Il-15* was considerably weaker in C2C12 myotubes treated with *Vdr*-specific siRNA. Vdr-KO mice showed significantly lower muscle *Il-*15 mRNA than WT mice. *Il-15Ra* mRNA and Il-15/Il-15Rα protein abundance were unaffected by 1,25(OH)_2_D_3_-treatment or VDR functionality, and Cyp27b1 activity is not required for 25(OH)D_3_-mediated *Il-15* gene expression.

The results provide evidence for a regulatory role of hydroxyvitamin D_3_ metabolites on the Il-15 synthesis in skeletal muscle cells, which is largely mediated by the VDR.

## Highlights

• Dose-dependent *Il-*15 mRNA increase in C2C12 myotubes by 1,25(OH)_2_D_3_ or 25(OH)D_3_ treatment.
• 1,25(OH)_2_D_3_ moderately increases the relative Il-15 protein amount in C2C12 myotubes.
• 1,25(OH)_2_D_3_ moderately increases the muscular *Il-*15 mRNA *in vivo*.
• 1,25(OH)_2_D_3_-mediated regulation of muscle *Il-*15 mRNA depends on the VDR.
• Reduced muscle *Il-*15 mRNA abundance in Vdr-KO mice compared to WT mice.

## 1 Introduction

The number of studies demonstrating extra-skeletal effects of vitamin D metabolites is steadily increasing [1,2], confirming the central importance of vitamin D for the maintenance of human health [3]. Particularly noticeable are the effects of vitamin D metabolites on the innate and adaptive immune response [4,5] and on muscle morphology and physiology [6].

Both immune cells and muscle cells express CYP enzymes such as CYP27B1 [[7], [8], [9], [10]], which catalyzes the formation of 1,25-dihydroxyvitamin D_3_ (1,25(OH)_2_D_3_) from 25-hydroxyvitamin D_3_ (25(OH)D_3_) [8,[11], [12], [13]]. Both types of cells also express the vitamin D receptor (VDR) [8,10,[13], [14], [15]], the nuclear factor that mediates the 1,25(OH)_2_D_3_ effects [16]. Because both types of cells display factors important in the regulation of vitamin D metabolism, their regulation by vitamin D metabolites becomes plausible. The active vitamin D_3_ hormone 1,25(OH)_2_D_3_, for example, has been shown to regulate the proliferation and development of immune cells, in particular natural killer cells, T cells, and B cells and inflammatory cytokine expression [4,5]. In muscle cells it improves muscle mass and strength, but also influences fiber remodeling, anabolic and catabolic processes [6,13]. Consequently, studies have shown close correlations between a low vitamin D status and the prevalence of immune pathologies such as autoimmune diseases [5] or reduced muscle mass [6,13,17].

Besides the fact, that the immune system and the musculature depend on vitamin D, both tissues show a strong mutual relationship, called “muscle-immune-crosstalk”. Hereby, muscle-derived signaling molecules, termed as myokines, affect downstream regulation of the immune system and the musculature [[18], [19], [20]]. Several immunologically active myokines, including interleukin-6 (IL-6) and IL-7, have already been found to be central regulators of the muscle-immune axis [[20], [21], [22]]. In the last years, IL-15 has gained increasing interest due to its pivotal role in the regulation of immune function, muscle homeostasis but also cancer [19,20,22,23].

IL-15 is an interesting and effective molecule that can regulate the development and maintenance of immune cells [22], particularly the proliferation, activation, and distribution of NK cells, T cells, and B cells [24]. In addition to its important immunoregulatory function, IL-15 also acts in a para- and autocrine manner on myogenesis [25], muscle mass [26], muscle regeneration [27], and energy metabolism of muscle cells [28]. Remarkably, IL-15 is expressed in a variety of tissues, including several immune cell types, as well as heart, lung, and placenta [23,[28], [29], [30]]. Remarkably, the muscle shows a particularly high expression of IL-15, which explains why IL-15 is called a myokine [28,31,32]. In muscle, IL-15 mRNA stability, intracellular trafficking, transmembrane presentation, and secretion are regulated by its high-affinity receptor IL-15Rα [30,33,34]. IL-15 mediates its signals mainly in a cell contact-dependent manner by binding to the IL-15Rα, forming a membrane-bound Il-15/IL-15Rα complex. which is presented to neighboring cells expressing IL-2/IL-15Rβ and the common γc chain [[35], [36], [37], [38]]. In addition, signal transmission can also take place endocrine at more distant tissues via the secreted IL-15/IL-15Rα complex and by binding to the β and γ subunits of the IL-2 receptor on the cell surface of its target tissue (IL-2Rβ/γc), activating the JAK/STAT signaling pathways [23,28,30,33]. Interestingly, physical exercise stimulates the IL-15 expression and secretion [21,31,32,39], while aging decreases it [32,40,41]. The role of other regulators of muscle IL-15, and IL-15Rα in particular, is largely unknown.

Here, we hypothesize that vitamin D may play a role in the regulation of muscle IL-15 synthesis and of its receptor IL-15Rα, as it has already been described as a regulator of many other cytokines and myokines [4,[42], [43], [44]].

## 2 Material & methods

### 2.1 Cell culture and treatments

Murine C2C12 muscle myoblasts (CRL-1772; ATCC, Manassas, VA, USA) were cultured following standard protocols [45]. Briefly, C2C12 myoblasts were grown under proliferative conditions in growth medium consisting of Dulbecco's modified Eagle's medium (DMEM) supplemented with 10 % fetal bovine serum (FBS), 100 U/ml penicillin, and 100 μg/ml streptomycin (all reagents from Gibco, Life Technologies, Darmstadt, Germany). Myoblasts between the 10th and 20th in-house passages were used for experiments. For treatments, 1 × 10^5^ cells were seeded per well in 6-well plates and cultured in growth medium for 48 h. Differentiation into C2C12 myotubes was initiated after cells reached 80–90 % confluence by replacing the growth medium with differentiation medium containing DMEM supplemented with 2 % horse serum (Sigma‒Aldrich, Schnelldorf, Germany), 50 U/ml penicillin, and 50 μg/ml streptomycin. At day 3 of differentiation, myotubes were treated with vitamin D_3_, or 25(OH)D_3_ (both 25–100 nM; both from Sigma-Aldrich, Germany) or 1,25(OH)_2_D_3_ (0.1–100 nM; Tocris, Bristol, UK) for 24 h, with ethanol used as vehicle control.

For *Vdr* silencing, 1 × 10^5^ C2C12 myoblasts were seeded and cultured for 48 h in growth medium. After 48 h, myoblasts were transfected with 100 nM ON-TARGETplus non-targeting control siRNA (D-001810-10-20) or 100 nM ON-TARGETplus Mouse SMARTpool *Vdr* siRNA (L-058923-01-0020) using 7.5 μl of DharmaFECT 1 transfection reagent (all reagents from Dharmacon, Lafayette, CO, USA) in antibiotic-free differentiation medium. After 72 h of silencing, the formed *Vdr*-specific or non-targeting siRNA-transfected myotubes were treated with 10 nM 1,25(OH)_2_D_3_ or ethanol vehicle for additional 6 h in complete differentiation medium.

For the quantification of the cellular Il-15 protein abundance 3 × 10^5^ C2C12 myoblasts were seeded in 25 cm^2^ cell culture flasks and cultured under proliferative conditions and differentiated after 48 h into C2C12 myotubes as described above. At day 3 of differentiation, myotubes were treated with either 100 nM of 25(OH)D_3_ or 1,25(OH)_2_D_3_ for 24 h, with ethanol as vehicle control treatment. After treatment for 24 h, C2C12 myotubes were lysed using a standard lysis buffer, containing Halt™ protease and phosphatase inhibitor cocktail (Thermo Fisher Scientific, Germany) and stored on ice for 30 min before centrifugation at 13,000 rpm at 4 °C for 10 min. The supernatant was then removed, quantified by Bradford protein determination, and stored at – 20 °C until Western blot analysis.

For the quantification of the cellular protein amount of Il-15/Il-15Rα complexes, 2.5 × 10^5^ C2C12 myoblasts were seeded in 25 cm^2^ cell culture flasks in growth medium under proliferative conditions and differentiation was induced after 48 h as described above. At day 3 of differentiation, myotubes were treated with 10 nM 1,25(OH)_2_D_3_ or ethanol vehicle for 24 h. After treatment, cells were washed once with PBS (Sigma-Aldrich, Germany), then covered with 1 ml new PBS and scraped with a cell scraper. The cell suspension was removed and centrifuged for 4 min at 2000 rpm. The supernatant was removed and the cell pellet was resuspended in 100 μl of non-denaturing lysis buffer (150 mM NaCl, 1 mM Na_2_EDTA, 1 mM EGTA (all from Roth, Karlsruhe, Germany), 20 mM Tris, and 1 % Triton X-100; both from AppliChem, Darmstadt, Germany) containing Halt™ protease and phosphatase inhibitor cocktail (Thermo Fisher Scientific, USA) and stored on ice for 30 min before centrifugation at 13,000 rpm at 4 °C for 5 min. The supernatant was then removed, quantified by Bradford protein determination, and stored at – 20 °C until analysis.

### 2.2 Animals and treatments

To understand the role of CYP27B1 in muscle tissues compared to the classical tissues, gastrocnemius and soleus muscle samples were compared with kidney, liver, and spleen from four 32-to 40-week-old wild-type (WT) mice (one female and three males) of the B6.129-Cyp27b1^tm1Star^/Apb mouse strain [46]. To analyze the role of CYP27B1 in muscle *Il-15* gene expression, gastrocnemius muscles from the four WT and from four 32-to 40-week-old Cyp27b1-knockout (KO) mice (three females and one male) with the same genetic background as the WT mice were used. The mice used were offspring of heterozygote mice received as a gift from Dr. René St-Arnaud from McGill University, Montreal, Canada. WT mice were fed a commercial standard diet (ssniff Spezialitäten GmbH, Soest, Germany) and KO mice a standard rescue diet with 2 % calcium, 1.25 % phosphorus, 20 % lactose, and supplemented with 2200 IU vitamin D_3_/kg *ad libitum*. For *Il-*15 mRNA and Cyp27b1 protein analysis, gastrocnemius and soleus muscles as well as kidney, liver, and spleen were collected, immediately snap frozen in liquid nitrogen, and stored at −80 °C until analysis (Approval No. 203.m42502-2-1764 MLU).

To investigate the importance of the VDR for muscle Il-15 and Il-15Rα synthesis, plasma and gastrocnemius muscle samples from eight 22- to 41-week-old Vdr-knockout (Vdr-KO, two females and six males) mice and eight 18- to 40-week-old wild-type (WT) mice (three females and five males) were used. C57Bl/6N-Vdr^em1Fau^ mice were generated at the Friedrich-Alexander-University Erlangen-Nürnberg (FAU) via endonuclease-mediated mutation 1 (CRISPR and first mutation created). In brief, 2 gRNAs flanking exon 2 of the *VDR* gene were coinjected with Cas9 nuclease into C57BL/6 N fertilized eggs to generate a deletion of exon 2 by DNA repair. Vdr-KO mice were fed a commercial rescue diet (ssniff Spezialdiäten GmbH, Germany). For *Il-15* and *Il-15Ra* gene expression analysis, gastrocnemius muscles were collected, immediately snap frozen in liquid nitrogen, and stored at −80 °C until analysis. For analysis of the Il-15/Il-15Rα protein complex, collected plasma was used (Approval No. RUF-55.2.2-2532-2-1625).

To test whether 1,25(OH)_2_D_3_ is capable of acute regulation of muscle *Il-15* and *Il-15Rα* gene expression, snap-frozen samples of gastrocnemius muscle from rats were used for analysis from a previous study [47]. Briefly, these 8- to 11-week-old female Sprague-Dawley rats (*n* = 16) maintained on a standard chow diet (ssniff Spezialdiäten GmbH, Germany), received either a single intraperitoneal injection of 1,25(OH)_2_D_3_ (*n* = 9, 0.5 μg/kg body weight, Decostriol®, mibe GmbH Arzneimittel, Brehna, Germany) or an intraperitoneal applied placebo (*n* = 7) 12 h before sacrifice (Approval No. 33.12-42502-04-15 1995). All animal experiments were performed according to EU Directive 2010/63/EU for animal experiments.

### 2.3 RNA isolation and quantitative real-time PCR

Total RNA was extracted from gastrocnemius muscles of mice and rats as well as C2C12 myotubes using TriFast reagent (Peqlab, Germany) following the manufacturer's instructions. For cDNA synthesis, 1.2 μg of extracted total RNA was reverse-transcribed using the GoScript™ Reverse Transcription System with random primers (both Promega, Mannheim, Germany). The thermal cycling conditions were: 25 °C for 5 min, 42 °C for 1 h, and 70 °C for 15 min. Quantitative reverse-transcription real-time PCR (qRT‒PCR) was performed on a Rotor-Gene Q Cycler (Qiagen, Germany) using GoTaq qPCR Master Mix (Promega, Germany). The PCR conditions involved initial denaturation at 95 °C for 3 min, followed by cycling at 95 °C for 10 s, primer-specific annealing temperature for 30 s, and elongation at 72 °C for 30 s. C_T_ values of *Gapdh* were statistically compared between groups to ensure that *Gapdh* was stable and independent of the experimental conditions and a reliable reference gene. The relative mRNA expression levels of *Il-15, Il-15Ra,* and *Vdr* were determined and normalized to *Gapdh* expression in the same cDNA sample. Relative quantification of *Il-15, Il-15Ra, Cyp24a1,* and *Vdr* gene expression was performed by the 2^-ΔCT^ (ΔC_T_ = C_T_ [target gene] – C_T_ [reference gene]) method [48]. The following primers (5'→ 3′ orientation) were used.

**Table:** 

Gene	Species	Sequence	Annealing [°C]	Cycles
		F: 5′-3′		
		R: 5′-3′		
*Cyp24a1*	Mouse	CGTTCTGGGTGAATACACGCTAC	58	40
		TTCGGGTCTAAACTTGTCAGCATC		
*Cyp24a1*	Rat	CTCGGACCCTTGACAAACCA	60	45
		CCGAATGGGAGATGAGCGAA		
*Gapdh*	Mouse	GGTGAAGGTCGGTGTGAACG	58	20
		CTCGCTCCTGGAAGATGGTG		
*Gapdh*	Rat	AGTTCAACGGCACAGTCAAG	59	30
		TACTCAGCACCAGCATCAC		
*Il-15*	Mouse	TTCATGTCTTCATTTTGGGC	59	35
		TCTCCAGGTCATATCTTACATC		
*Il-15*	Rat	TTCATGTCTTCATTTTGGGC	59	35
		TCTCCAGGTCATATCTTACATC		
*Il-15Ra*	Mouse	AGGATAACAGAGATTTCTCCC	57	35
		CACAGTCATTGGTACTGTTTC		
*Il-15Ra*	Rat	AAAAGAGCCAGAAGCTTTATC	60	35
		ATTGTTGTTGCAAGAGTGG		
*Vdr*	Mouse	CCCCCACACCCCCACAAC CACATCTCCACCCCACTTACCAAT	60	35

### 2.4 Enzyme-linked immunosorbent assay (ELISA)

The cellular protein amount of Il-15/Il-15Rα complexes was determined in 100 μg of the C2C12 myotube cell lysate and the plasma concentration of Il-15/Il-15Rα protein complex was analyzed in mice and using a commercial ELISA (Mouse IL-15/IL-15Rα; Invitrogen, Thermo Fisher Scientific, Waltham, MA, USA) according to the manufacturer's protocol.

### 2.5 Western blotting

To analyze relative cellular Il-15 protein abundance in C2C12 myotubes after treatment with either 25(OH)D_3_ or 1,25(OH)_2_D_3_, 50 μg of whole cell lysate was used in a standard Western blot protocol and nitrocellulose membranes were incubated overnight with the following antibodies at 1:1000 dilution at 4 °C: IL-15 (ab273625); Abcam, Cambridge, UK) and GAPDH (#5174; Cell Signaling Technology, Danvers, MA, USA), followed by incubation with secondary HRP-conjugated antibodies at a 1:2000 dilution at room temperature for 2 h: anti-rabbit IgG (#7074; Cell Signaling Technology, USA).

To examine Cyp27b1 expression in muscle tissues in comparison to tissues with well-known CYP27B1 expression, gastrocnemius and soleus muscle samples from mice and differentiated C2C12 myotubes as well as kidney, liver, and spleen were lysed using a standard lysis buffer, containing Halt™ protease and phosphatase inhibitor cocktail (Thermo Fisher Scientific, Germany) and quantified by Bradford protein determination. Subsequently, 50 μg of total protein lysate per sample was used in a standard Western blot protocol using the following antibodies at 1:1000 dilution for overnight incubation of nitrocellulose membranes at 4 °C: CYP27B1 (ab206655; Abcam, UK), GAPDH (#5174; Cell Signaling Technology, USA), followed by incubation with secondary HRP-conjugated antibodies at a 1:2000 dilution at room temperature for 2 h: anti-rabbit IgG (#7074; Cell Signaling Technology, USA). Protein band analysis of these Western blot experiments were performed using ECL detection reagent (GE healthcare-Amersham, Amersham, UK) and the Syngene G:BOX Chemi XX6 (VWR, Dresden, Germany) detection system. Protein band intensities of Cyp27b1 and Il-15 were normalized to Gapdh as loading control.

### 2.6 Statistics

The data are presented as arithmetic mean ± SEM, where *n* represents the number of independent experiments conducted. Normality of data distribution was assessed using the *Shapiro‒Wilk* test. For non-normally distributed data, all other comparisons were analyzed with a non-parametric test in the same experimental design. For comparisons between two groups, unpaired *Student's t*-test or *Mann-Whitney U* test (for non-normally distributed data) was used. Comparisons involving more than two treatments were analyzed using one-way *ANOVA* followed by *Tukey's* multiple comparison test (for normally distributed data) or the *Kruskal‒Wallis* test followed by *Dunn's* multiple comparison test (for non-normally distributed data). Differences were considered significant at *p* < 0.05.

## 3 Results

### 3.1 Effects of vitamin D metabolites on Il-15 and Il-15Rα synthesis in C2C12 myotubes

**Fig. 1:**
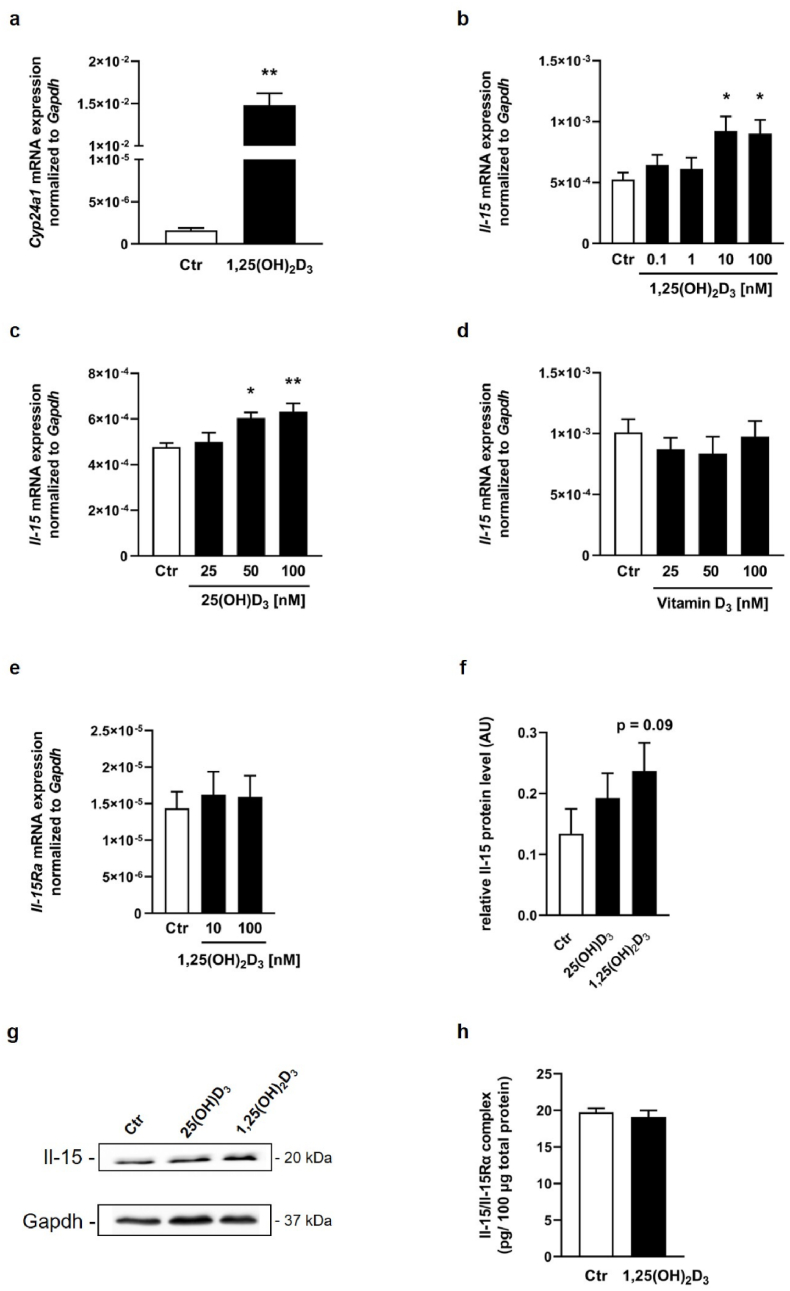
Effects of vitamin D metabolites on Il-15 and Il-15Rα synthesis in C2C12 myotubes Arithmetic means ± SEM of relative Cyp24a1 (**a**) *Il-15* (**b**–**d**) or *Il-15Ra* (**e**) mRNA abundance (normalized to *Gapdh*) or cellular Il-15 (**f**) or Il-15/Il-15Rα protein complexes (**h**) in murine differentiated C2C12 myotubes treated with increasing concentrations (black bars as indicated) of either 1,25(OH)_2_D_3_ (**a**: *n* = 6, 100 nM; **b**: *n* = 10; **e**: *n* = 7; **f**: *n* = 5 h, 100 nM; **h**: *n* = 11, 10 nM) or 25(OH)D_3_ (**c**: *n* = 9; **f**: *n* = 5, 100 nM) or vitamin D_3_ (**d**: *n* = 5) or corresponding EtOH vehicle control (white bars) alone for 24 h. **g** Original representative Western blot demonstrating Il-15 and Gapdh protein abundance in C2C12 myotubes after treatment with 100 nM of either 25(OH)D_3_, 1,25(OH)_2_D_3_, or EtOH alone for 24 h ∗*p* < 0.05 and ∗∗*p* < 0.01 indicates significant differences from control. AU, arbitrary units; Ctr, control; 1,25(OH)_2_D_3_, active vitamin D_3_ hormone, calcitriol; 25(OH)D_3_, 25-hydroxyvitamin D_3_ (**a**, **f, h**: Mann-Whitney *U* test; **b**–**e**: *Kruskal-Wallis* with *Dunn's post-hoc* test.

In order to evaluate the efficacy of the highly potent 1,25(OH)_2_D_3_ in C2C12 myotubes, the mRNA abundance of its target gene *Cyp24a1* was analyzed. As shown in Fig. 1a, treatment with 1,25(OH)_2_D_3_ strongly increased *Cyp24a1* mRNA abundance in C2C12 myotubes compared to control cells. Next, to investigate whether vitamin D metabolites can directly stimulate the expression of the *Il-15* gene in muscle cells, differentiated mouse C2C12 myotubes were treated with increasing concentrations of 1,25(OH)_2_D_3_, 25(OH)D_3_, or vitamin D_3_ for 24 h. Here, we found a dose-dependent increase in the mRNA abundance of *Il-*15 mRNA in C2C12 myotubes treated with 1,25(OH)_2_D_3_ and 25(OH)D_3_ for 24 h compared to the control cells (Fig. 1b and c). That increase in *Il-*15 mRNA abundance was accompanied by moderately higher Il-15 protein levels in C2C12 myotubes after treatment with 1,25(OH)_2_D_3_ (Fig. 1f and g). Treatment with 25(OH)D_3_ (Fig. 1f and g) also tended to increase relative Il-15 protein abundance, but without any statistical significance, indicating the stronger effect of 1,25(OH)_2_D_3_. Conversely, treatment with vitamin D_3_, even at high concentrations, did not affect *Il-*15 mRNA expression in C2C12 myotubes (Fig. 1 d). In addition, it was tested whether 1,25(OH)_2_D_3_, can also regulate *Il-15Ra* mRNA. As depicted in Fig. 1 e, we found no effect of 1,25(OH)_2_D_3_-regulation on the mRNA abundance of *Il-15Ra* in C2C12 myotubes after treatment for 24 h. Since 1,25(OH)_2_D_3_ mediated the strongest biological effect, we finally investigated the cellular levels of Il-15/Il-15Rα protein complexes, but we found no differences between C2C12 myotubes treated with 1,25(OH)_2_D_3_ in comparison to the control cells (Fig. 1 h).

### 3.2 Administration of a single intraperitoneal 1,25(OH)_2_D_3_ application upregulated *Il-*15 mRNA in rat muscles

**Fig. 2:**
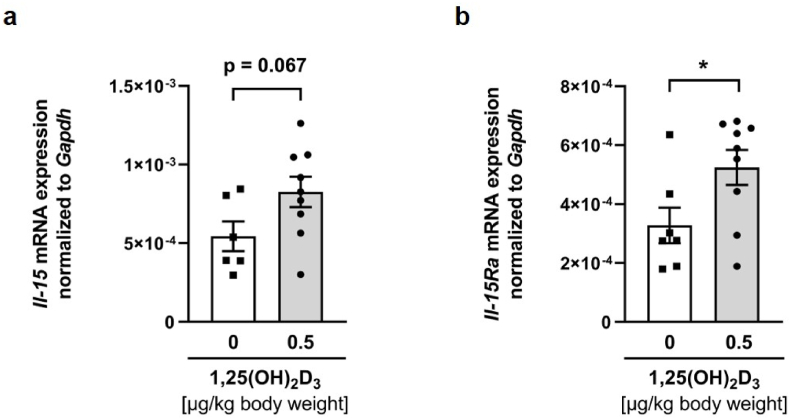
Administration of a single intraperitoneal 1,25(OH)_2_D_3_ application upregulated *Il-*15 mRNA in rat muscles Scatter dot plots and arithmetic means ± SEM of relative *Il-15* (**a**) or *Il-15Ra* (**b**) mRNA abundance (normalized to *Gapdh*) in gastrocnemius muscle of rats treated with either a single intraperitoneal injection of 1,25(OH)_2_D_3_ 12 h before sacrifice (0.5 μg/kg body weight; **a**, **b**: *n* = 6–9) or the corresponding placebo. ∗p < 0.05 indicates significant differences from placebo control. 1,25(OH)_2_D_3_, active vitamin D_3_ hormone, calcitriol (**a**, **b**: unpaired Student's *t*-test).

To determine whether 1,25(OH)_2_D_3_ is also capable of stimulating *Il-*15 mRNA abundance *in vivo*, rat muscle samples from a previous study [47] were used and analyzed by qRT-PCR. In this study, rats were treated with either 0.5 μg/kg body weight or placebo by intraperitoneal injection 12 h before sacrifice. To test whether this intraperitoneal injection of 1,25(OH)_2_D_3_ is an effective treatment to reach the muscles and be absorbed, we first analyzed the mRNA abundance of *Cyp24a1* in the gastrocnemius muscles of six placebo and nine 1,25(OH)_2_D_3_-treated rats. Here we observed that *Cyp24a1* mRNA abundance was below the detection level in gastrocnemius muscles of placebo-treated rats, whereas *Cyp24a1* mRNA was detectable in six out of nine rats 12 h after intraperitoneal injection of 1,25(OH)_2_D_3_. This demonstrates the efficacy of the treatment and the ability of this metabolite to reach the gastrocnemius muscle and its muscular uptake. Having shown that 1,25(OH)_2_D_3_ is taken up by muscle, we analyzed whether a single intraperitoneal application of 1,25(OH)_2_D_3_ also regulates muscle *Il-*15 mRNA abundance *in vivo*. Here, in agreement with our previous results, we observed a moderate increase in *Il-*15 mRNA abundance in gastrocnemius muscles of rats treated with a single intraperitoneal injection of 1,25(OH)_2_D_3_ 12 h before sacrifice compared to placebo-treated rats (*p* = 0.067; Fig. 2 a). Interestingly, in contrast to the data obtained from C2C12 myotubes, the gastrocnemius muscle of 1,25(OH)_2_D_3_-treated rats showed a significantly higher *Il-15Ra* mRNA abundance than the placebo-treated controls (Fig. 2 b).

### 3.3 Muscle Il-15 synthesis is independent of Cyp27b1 activity

**Fig. 3:**
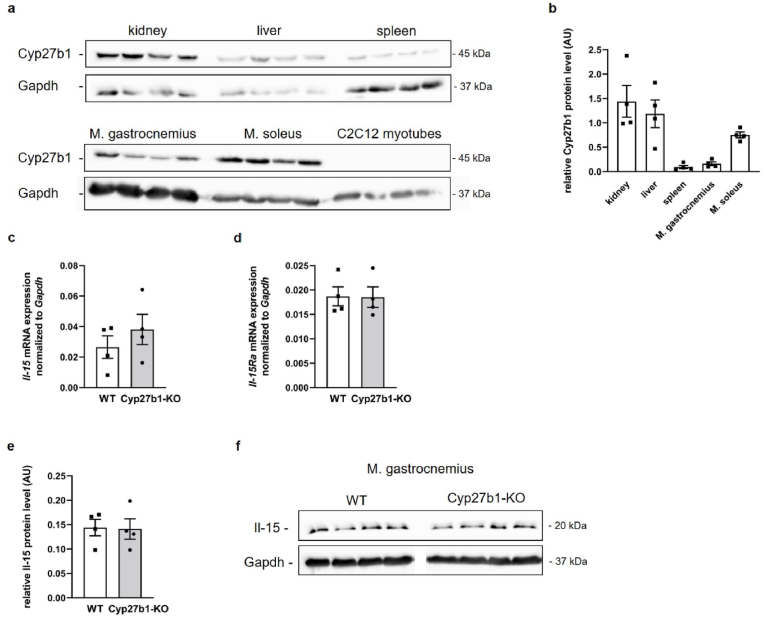
Muscle Il-15 synthesis is independent of Cyp27b1 activity Original Western blots (**a**) demonstrating Cyp27b1 and Gapdh protein abundance in 50 μg protein lysate from kidney, liver, spleen, gastrocnemius and soleus muscles (M.) of wild-type mice (*n* = 4) and differentiated C2C12 myotubes (*n* = 4). Scatter dot plots and arithmetic means ± SEM of densitometric analysis of relative (**b**) Cyp27b1 protein abundance normalized to loading control Gapdh in kidney, liver, spleen, M. gastrocnemius and M. soleus of wild-type mice. Scatter dot plots and arithmetic means ± SEM of relative *Il-15* (**c**) or *Il-15Ra* (**d**) mRNA or (**e**) Il-15 protein abundance (normalized to Gapdh) in gastrocnemius muscle of wild-type (WT) or Cyp27b1 knockout (Cyp27b1-KO) mice. Original Western blots (**f**) demonstrating Il-15 and Gapdh protein abundance in 50 μg protein lysate from M. gastrocnemius of WT or Cyp27b1-KO mice. AU, arbitrary units. (**c**–**e**: *n* = 4; Unpaired Student's *t*-test).

Before we investigated the role of CYP27B1 in the regulation of Il-15 in muscle, we analyzed the extent of Cyp27b1 expression in muscle and compared it with Cyp27b1 protein expression in 1,25(OH)_2_D_3_-producing organs such as kidney, liver, and spleen of wild-type mice. Notably, as shown in Fig. 3a and b, Cyp27b1 protein expression in soleus muscle was higher than that in spleen and almost as high as that in liver and kidney, whereas Cyp27b1 protein levels in gastrocnemius muscle were similar to that in spleen, demonstrating the ability of skeletal muscle to produce 1,25(OH)_2_D_3_. Interestingly, Cyp27b1 protein abundance was higher in soleus than in gastrocnemius muscle, indicating differences within muscle tissues (Fig. 3a and b). Notably, Cyp27b1 protein expression was not detectable in whole cell lysate obtained from differentiated C2C12 myotubes (Fig. 3 a). This prompted us to investigate whether the increase of *Il-*15 mRNA in 25(OH)D_3_-treated C2C12 myotubes was independent of the conversion of 25(OH)D_3_ to 1,25(OH)_2_D_3_. To test this, Il-15 mRNA and protein expression was measured in gastrocnemius muscles of *Cyp27b1*-knockout (KO) mice, which have undetectable 1,25(OH)_2_D_3_ level [46], compared to their wild-type (WT) controls. Remarkably, *Il-*15 mRNA abundance (Fig. 3 c) and Il-15 protein expression (Fig. 3e and f) was similar between *Cyp27b1*-KO and WT mice, suggesting that 25(OH)D_3_ could directly regulate Il-15 synthesis without 1α-hydroxylation by CYP27B1 enzyme. In line with the *in vitro* data was the finding that *Il-15Ra* mRNA abundance in gastrocnemius muscles did not differ between *Cyp27b1*-KO and WT mice (Fig. 3 d).

### 3.4 Muscle *Il-*15 mRNA expression differs between sexes in mice

**Fig. 4:**
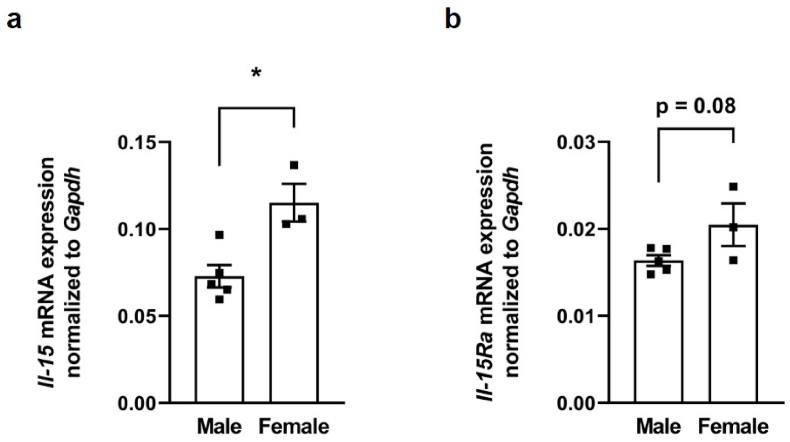
Muscle *Il-*15 mRNA expression differs between sexes in mice Scatter dot plots and arithmetic means ± SEM of relative *Il-15* (**a**) or *Il-15Ra* (**b**) mRNA abundance (normalized to *Gapdh*) in gastrocnemius muscle of wild-type (WT) mice (**a**, **b**: *n* = 3–5). ∗*p* < 0.05 indicates significant differences between males and females. (**a**, **b**: Unpaired Student's *t*-test).

Since it has been previously reported that muscle Il-15 protein expression levels in mice are sex-dependent, we also analyzed whether this effect could be observed in our gastrocnemius muscle samples from WT mice. Interestingly, and in line with these previous data, *Il-*15 mRNA abundance was significantly higher in gastrocnemius muscle of female mice compared to male mice (Fig. 4 a). In addition, *Il-15Ra* mRNA abundance was also moderately higher (*p* = 0.08) in gastrocnemius muscles of female mice compared to male mice (Fig. 4 b). The sample size of female Vdr-KO mice was too small to analyze whether this effect could also be observed in Vdr-KO mice.

### 3.5 1,25(OH)_2_D_3_-stimulated *Il-*15 mRNA expression depends on the VDR

**Fig. 5:**
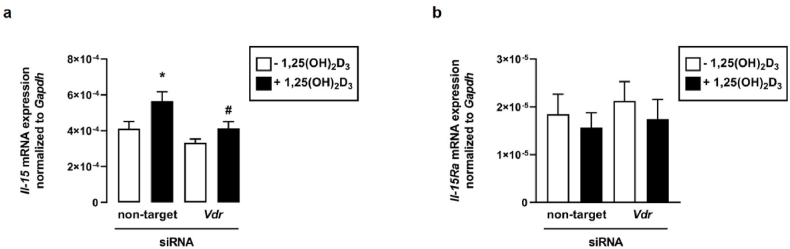
1,25(OH)_2_D_3_-stimulated *Il-*15 mRNA expression depends on the VDR Arithmetic means ± SEM of relative *Il-15* (**a**: *n* = 8) or *Il-15Ra* (**b**: *n* = 5) mRNA abundance (normalized to *Gapdh*) in murine differentiated C2C12 myotubes incubated with non-targeting or *Vdr*-specific siRNA (**a**, **b** 100 nM, 72 h; *n* = 8) in the absence (white bars) or presence (black bars) of 1,25(OH)_2_D_3_ (**a**, **b**: 10 nM, 6 h). Treatment with *Vdr*-specific siRNA resulted in a 53 % reduction in relative *Vdr* mRNA abundance. ∗*p* < 0.05 indicates significant differences from control; ^#^*p* < 0.05 indicates significant differences from the absence of *Vdr*-specific siRNA (2nd vs. 4th bar). Vdr, vitamin d receptor; 1,25(OH)_2_D_3_, active vitamin D_3_ hormone, calcitriol (**a**, **b**: one-way *ANOVA* with *Tukey's post-hoc* test).

To investigate whether the 1,25(OH)_2_D_3_ effect on *Il-*15 mRNA abundance was mediated through the VDR, we treated the C2C12 myotubes with and without *Vdr*-specific siRNA in the presence or absence of 1,25(OH)_2_D_3_. The efficiency of reducing *Vdr* mRNA abundance following the treatment with *Vdr*-specific siRNA was 53 % (p < 0.05). The data showed a significant reduction of the 1,25(OH)_2_D_3_-mediated upregulation of *Il-15* in C2C12 myotubes treated with *Vdr*-specific siRNA compared to the non-targeting siRNA treatment (Fig. 5 a). This finding indicates the role of VDR in the mediation of the 1,25(OH)_2_D_3_ effects on *Il-15*. In line with our previous results, showing no effect of 1,25(OH)_2_D_3_ treatment on *Il-15Ra* mRNA abundance in C2C12 myotubes, neither the treatment with non-targeting or *Vdr*-specific siRNA alone or in combination with 1,25(OH)_2_D_3_ showed any influence on the *Il-15Ra* mRNA abundance in C2C12 myotubes (Fig. 5 b).

### 3.6 Muscle *Il-*15 mRNA expression is regulated by the VDR

**Fig. 6:**
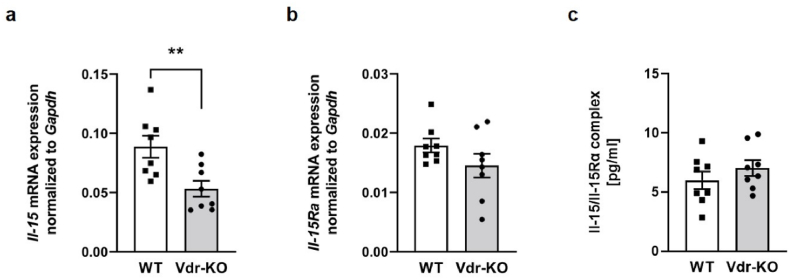
Muscle *Il-*15 mRNA expression is regulated by the VDR Scatter dot plots and arithmetic means ± SEM of relative *Il-15* (**a**) or *Il-15Ra* (**b**) mRNA abundance (normalized to *Gapdh*) in gastrocnemius muscle or Il-15/Il-15Rα protein complexes (**c**) in plasma of wild-type (WT) or vitamin D receptor knockout (Vdr-KO) mice (**a**–**c**: *n* = 8). ∗∗*p* < 0.01 indicates significant differences from WT mice. (**a**,**b**,**c**: Unpaired Student's *t*-test).

Because we identified VDR as the relevant transcription factor for the 1,25(OH)_2_D_3_-stimulated *Il-*15 mRNA abundance in C2C12 myotubes, we hypothesized that Vdr-KO mice may have a lower *Il-*15 mRNA abundance in their gastrocnemius muscles compared to their WT counterparts. Indeed, the results demonstrated lower muscle *Il-*15 mRNA levels in the Vdr-KO group compared to the WT controls (Fig. 6 a). Moreover, consistent with our *in vitro* data, Vdr-KO mice did not differ in muscle *Il-15Ra* mRNA abundance (Fig. 6 b) and circulating plasma Il-15/Il-15Rα complexes compared to the WT control mice (Fig. 6 c).

## 4 Discussion

Our study demonstrates that hydroxylated metabolites of vitamin D_3_, in particular 1,25(OH)_2_D_3_, and its transcription factor VDR are novel regulators of Il-15 production in rodent muscle cells.

The effect of 1,25(OH)_2_D_3_ was largely mediated through the VDR, as 1,25(OH)_2_D_3_-mediated induction of *Il-15* gene expression was reduced in C2C12 myotubes treated with *Vdr*-specific siRNA and muscle *Il-15* was strongly reduced in Vdr-KO mice compared to their WT controls.

Thus, our data clearly demonstrated the importance of muscle tissue for 1,25(OH)_2_D_3_-mediated induction of Il-15 mRNA and protein synthesis, which, along with monocytes, is one of the major IL-15 producing and secreting tissues compared to other IL-15 synthesizing tissues such as kidney, placenta, lung or heart [49].

Our results also show that 25(OH)D_3_ could stimulate *Il-15* gene expression, which was evidenced by the dose-dependent increase in the mRNA abundance of *Il-15* in C2C12 myotubes upon treatment with 25(OH)D_3_.We first assumed that this effect of 25(OH)D_3_ was caused by its conversion to 1,25(OH)_2_D_3_ because muscle C2C12 myotubes have been described to be able to express CYP27B1 [8]. However, current data do not show any Cyp27b1 protein expression in C2C12 myotubes, indicating that a further 1α-hydroxylation of 25(OH)D_3_ was not necessary for the observed stimulation of muscle *Il-*15 mRNA in C2C12 myotubes. This assumption was corroborated by the finding that the mRNA and protein expression of muscle Il-15 did not differ between Cyp27b1-KO mice, which have non-detectable 1,25(OH)_2_D_3_ levels [46], and WT mice. Some other studies also describe a transcriptional activity of 25(OH)D_3_ without the need for conversion to 1,25(OH)_2_D_3_ [50], also in muscle [51]. Thus, both pathways are potentially possible, muscle could use circulating 25(OH)D_3_ to store and locally convert it to 1,25(OH)_2_D_3_, thus becoming independent of renal 1,25(OH)_2_D_3_ production through local synthesis [10,52,53], or 25(OH)D_3_ can act directly in the absence of enzymatic conversion to 1,25(OH)_2_D_3_, as demonstrated by our data in Cyp27b1-KO mice.

The importance of the activation of vitamin D_3_ metabolites by hydroxylation to regulate *Il-15* gene expression, is further confirmed by our finding that vitamin D_3_ itself had no effect on *Il-*15 mRNA abundance in C2C12 myotubes. This result can be explained by the low or even absent expression of the necessary enzymes CYP27A1 or CYP2R1 in C2C12 myotubes and the resulting lack of conversion to 25(OH)D_3_ [54].

Interestingly, our results also showed differences between soleus and gastrocnemius muscle in the abundance of Cyp27b1 protein. Thus, the fiber type I-rich soleus muscle of WT mice had higher Cyp27b1 protein levels than the fiber type II-rich gastrocnemius muscle, which is consistent with data from other studies in mice [55]. This indicates a possible fiber type-specific CYP27B1 expression in muscle, suggesting a muscle type-specific vitamin D metabolism.

For activity, IL-15 must bind to its receptor, IL-15Rα. Data from other studies demonstrate that IL-15 protein expression is regulated by IL-15Rα [33] and that a lack of IL-15Rα co-expression results in a reduction of IL-15 synthesis and secretion [34]. The main function of IL-15Rα is the stabilization of the *IL-*15 mRNA and the binding of IL-15 to form a IL-15/IL-15Rα complex for transport into cell compartments, *trans*-presentation or secretion [34,37,56]. As we found moderately higher Il-15 protein levels in C2C12 myotubes after treatment with 1,25(OH)_2_D_3_, but no effects of 1,25(OH)_2_D_3_ treatment or intact VDR on the *Il-15Ra* mRNA abundance in cells and mice, it appears plausible that the cellular amount of Il-15/Il-15Rα complexes in C2C12 myotubes did not change with 1,25(OH)_2_D_3_-treatment and that the plasma Il-15/Il-15Rα concentrations were not different between Vdr-KO and WT mice. Thus, it can be speculated that Il-15Rα in muscle cells must be present in sufficient concentrations to result in higher secreted Il-15/Il-15Rα protein levels. This assumption is supported by other studies showing that primary myogenic cultures from *Il-15Rα* knockout mice were unable to secrete Il-15 protein after LPS stimulation, although *Il-*15 mRNA expression was also increased by LPS [57], and muscle-specific *Il-15Rα* knockout mice had lower circulating levels of Il-15 levels than control mice [56]. However, since IL-15 is capable to transmit its signal also in a cell contact-dependent manner by presenting membrane-bound IL-15-IL-15Rα complexes to neighboring cells [[36], [37], [38]], it could be speculated that the observed 1,25(OH)_2_D_3_-mediated increase in cellular Il-15 protein levels contributes to increased Il-15 *trans*-presentation and that 1,25(OH)_2_D_3_ mediates some of its muscle effects via Il-15 membrane-bound presentation on surrounding cells in muscle tissue. This would also explain the increased Il-15 protein expression with constant cellular Il-15/Il-15Rα complexes *in vitro* and circulating Il-15/Il-15Rα *in vivo*, as there is increased 1,25(OH)_2_D_3_-induced Il-15 presentation at the muscle cell surface rather than secretion of this complex into the circulation from the muscle cell. A more detailed investigation of this mechanism and possible downstream biological effects on surrounding tissues will be the subject of future studies.

In contrast, an unexpected finding of our study was the higher *Il-15Ra* mRNA expression in rats after intraperitoneal injection of 1,25(OH)_2_D_3_. Although we do not have an explicit explanation for this finding, we suspect that sex or species differences may be important factors in Il-15Rα expression, as discussed for IL-15 [56]. This is corroborated by our observations that *Il-15* and *Il-15Ra* mRNA expression was higher in the gastrocnemius muscles of female WT mice than in males, suggesting that *Il-15* and *Il-15Ra* expression is regulated in a sex-dependent manner. Thus, sex may also influence the metabolism and efficacy of 1,25(OH)_2_D_3_ and the expression of VDR, as described elsewhere [58], and may also explain the observed differences in *Il-15Ra* expression in this work.

However, relevant regulators of Il-15Rα must be identified in future studies to predict the conditions under which IL-15 will be *trans*-presented or secreted from the muscle cells.

Considering the multiple and important immunoregulatory and muscle functions of the myokine IL-15 [22,[24], [25], [26], [27]], which decisively influences the muscle-immune crosstalk, it is not surprising that a deterioration of skeletal muscle homeostasis, e.g. due to aging, which interestingly is associated with a reduction of IL-15 serum levels [40,41] but also with vitamin D inadequacy [59], also affects the function of the immune system and can lead to sarcopenia and immunosenescence, states of reduced quantity and function of IL-15 [22,59,60]. It is conceivable, that the observed Il-15-induction by 1,25(OH)_2_D_3_ in this study can possibly counteract or even reverse this decline in *IL-15* gene transcription and, by maintaining transcription, may beneficially counteract the progression of these pathophysiological age-related processes. Thus, it is assumed that pathologies associated with low vitamin D status, such as muscle weakness, increased muscle protein degradation, reduced muscle mass, strength, and myogenesis, decreased muscle mitochondrial energy production and mitochondrial biogenesis [17,59,[61], [62], [63]] may, at least in part, be caused by a reduction in IL-15 synthesis in muscle as IL-15 induces muscle growth, promotes oxidative energy metabolism and endurance, but decreases muscle proteolysis [[64], [65], [66]]. Moreover, low vitamin D is also associated with an inadequate immune response [67,68], which may also be a result of impaired IL-15 regulation. In line with this hypothesis, human data show that plasma 25(OH)D_3_ levels are positively correlated with plasma IL-15 levels in tuberculosis-infected patients [69] and also in men infected with COVID-19 [70]. Therefore, an adequate vitamin D supply may contribute to the maintenance of muscle homeostasis and immune system function via influencing IL-15 production in muscle [22].

However, the specific role of 1,25(OH)_2_D_3_-mediated increase in muscle IL-15/IL-15Rα *trans*-presentation or secretion needs to be addressed in further studies.

To conclude, hydroxylated metabolites of vitamin D_3_ can stimulate the mRNA and protein abundance of Il-15 in rodent muscle cells, an effect mediated, in a large part, by the VDR. IL-15 may therefore be a central signaling molecule in the vitamin D-mediated interaction between the immune system and muscle physiology.

## CRediT authorship contribution statement

**Franz Ewendt:** Conceptualization, Formal analysis, Investigation, Methodology, Visualization, Writing – original draft, Writing – review & editing. **Fabienne Drewitz:** Investigation. **Michael Althammer:** Resources, Investigation. **Cosima Eichler:** Investigation. **Corinna Brandsch:** Investigation. **Stefanie Brey:** Resources, Investigation. **Thomas H. Winkler:** Resources, Writing – review & editing. **Mirja R. Wilkens:** Resources, Investigation, Writing – review & editing. **René St-Arnaud:** Resources, Writing – review & editing. **Marina Kreutz:** Resources, Writing – review & editing. **Gabriele I. Stangl:** Conceptualization, Methodology, Resources, Supervision, Writing – original draft, Writing – review & editing.

## Funding

This work was supported by the 10.13039/501100001659Deutsche Forschungsgemeinschaft (DFG, German Research Foundation) project number 324392634 -TRR 221 project B12 (MK) and project Z02 (THW).

## Declaration of competing interest

The authors declare no competing interests.

## Data Availability

Data will be made available on request.
