# Supplementary material for: Vitamin D stimulates Il-15 synthesis in rodent muscle
Source: Biochem Biophys Rep. 2025 Jan 25;41:101925. doi: 10.1016/j.bbrep.2025.101925 (PMC11935148; doi:10.1016/j.bbrep.2025.101925)
Supplement: Multimedia component 1 [file mmc1.pdf]

Original western blot of Il-15 protein analysis in C2C12 myotubes

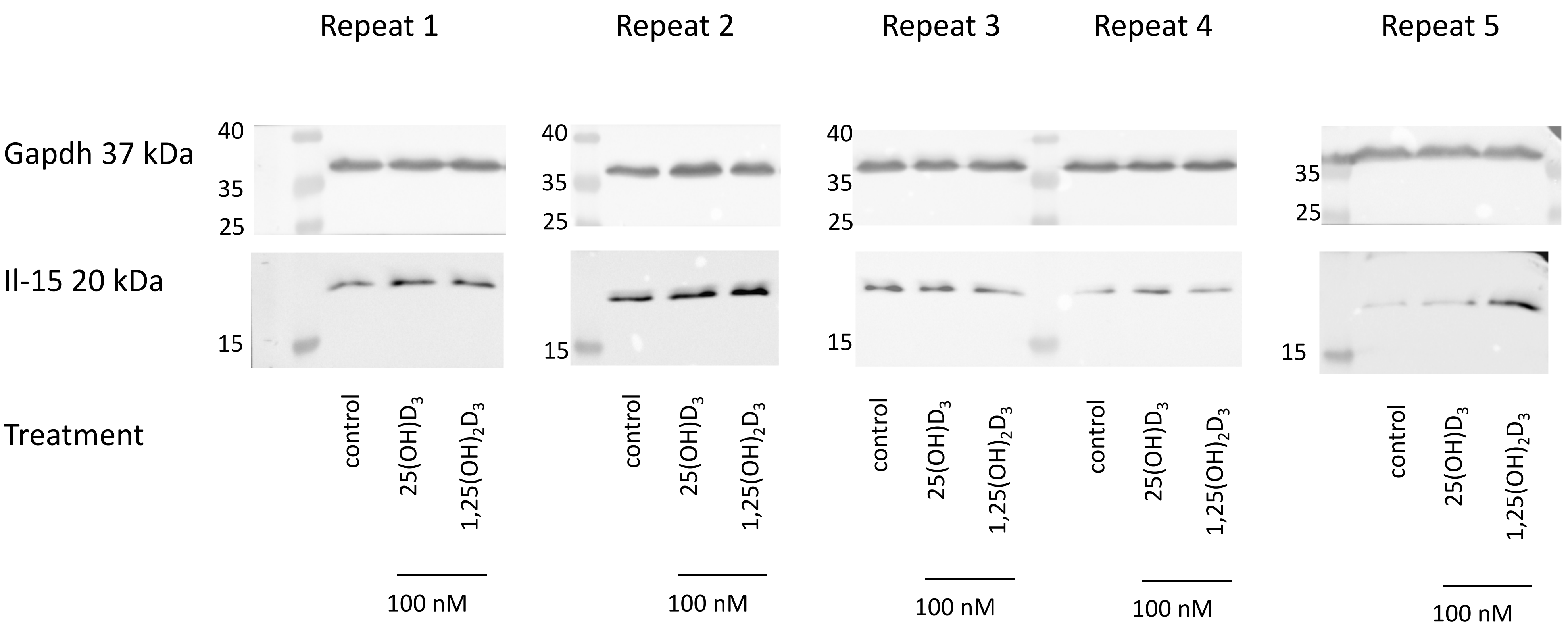

Original Western Blots with molecular marker corresponding to densitometric analysis of **Fig. 1 f** (*n*=5): Il-15 protein expression in C2C12 myotubes after treatment with either 25(OH)D_3 or 1,25$(OH)_2D_3$ for 24h compared to vehicle control. Whole blot was cutted over 40 kDa and under 25 kDa for Gapdh as loading control. For Il-15, whole blot was cutted under 25 kDa and under 15 kDa.
